# Investigating the 1-year decline in midbrain-to-pons ratio in the differential diagnosis of PSP and IPD

**DOI:** 10.1007/s00415-020-10327-2

**Published:** 2020-12-04

**Authors:** Silja Kannenberg, Julian Caspers, Lars Dinkelbach, Alexia-S. Moldovan, Stefano Ferrea, Martin Südmeyer, Markus Butz, Alfons Schnitzler, Christian J. Hartmann

**Affiliations:** 1 Institute of Clinical Neuroscience and Medical Psychology, Medical Faculty, Heinrich Heine University Düsseldorf, Universitätsstraße 1, 40225 Düsseldorf, Germany; 2 Department of Diagnostic and Interventional Radiology, Medical Faculty, University Hospital Düsseldorf, Moorenstraße 5, 40225 Düsseldorf, Germany; 3 Department of Neurology, Medical Faculty, University Hospital Düsseldorf, Moorenstraße 5, 40225 Düsseldorf, Germany; 4 Department of Neurology, Ernst Von Bergmann Hospital, Charlottenstraße 72, 14467 Potsdam, Germany

**Keywords:** Progressive supranuclear palsy, Idiopathic Parkinson’s disease, Midbrain-to-pons ratio, Atypical parkinsonism, MRI

## Abstract

**Background:**

A reliable measure of PSP-specific midbrain atrophy, the midbrain-to-pons ratio (MTPR) has been reported to support the differential diagnosis of progressive supranuclear palsy (PSP) from idiopathic Parkinson’s disease (IPD). Since longitudinal analyses are lacking so far, the present study aimed to evaluate the diagnostic value of the relative change of MTPR (relΔt_MTPR) over a 1-year period in patients with PSP, IPD, and healthy controls (HC).

**Methods:**

Midsagittal individual MRIs of patients with PSP (*n* = 15), IPD (*n* = 15), and healthy controls (HC; *n* = 15) were assessed and the MTPR at baseline and after 1 year were defined. The diagnostic accuracy of the MTPR and its relative change were evaluated using ROC curve analyses.

**Results:**

PSP-patients had a significantly lower MTPR at baseline (*M* = 0.45 ± 0.06), compared to both non-PSP groups (F (2, 41) = 62.82, *p* < 0.001), with an overall predictive accuracy of 95.6% for an MTPR ≤ 0.54. PSP-patients also presented a significantly stronger 1-year decline in MTPR compared to IPD (*p* < 0.001). Though predictive accuracy of relΔ_t__MTPR for PSP (*M* = − 4.74% ± 4.48) from IPD (*M* =  + 1.29 ± 3.77) was good (76.6%), ROC analysis did not reveal a significant improvement of diagnostic accuracy by combining the MTPR and relΔ_t__MTPR (*p* = 0.670). Still, specificity for PSP increased, though not significantly (*p* = 0.500).

**Conclusion:**

The present results indicate that the relΔ_t__MTPR is a potentially useful tool to support the differential diagnosis of PSP from IPD. For its relative 1-year change, still, more evaluation is needed.

## Introduction

The differential diagnosis of progressive supranuclear palsy (PSP) from Parkinson’s disease (IPD) is not trivial [1, 2]. At present, the clinical diagnosis of PSP is primarily based on identifying disease-specific symptoms, which may not have fully developed in early stages of the disease [3, 4]. Accordingly, misdiagnoses occur frequently due to a substantial overlap of symptoms [5]. Still, a more rapid progression and an overall poor prognosis in PSP underline the clinical need for objective biomarkers to facilitate early and precise diagnosis [6, 7].

As specific brain structures are known to be atrophic to different extents in different Parkinsonian diseases, disease-specific alterations detectable by structural magnetic resonance imaging (MRI) were suggested to support the diagnosis of PSP [8–13].

A hallmark, known to be highly specific of PSP, is midbrain atrophy [14, 15]. Hence, the so-called midbrain-to-pons ratio (MTPR) was introduced as a potential biomarker to distinguish between PSP and IPD and constitutes a reliable method to quantify PSP-specific midbrain atrophy [16–18]. Since PSP progresses considerably faster than IPD, PSP-specific rates of atrophy can potentially serve as biomarkers of the disease and support differential diagnosis [19, 20].

To substantiate this notion, we aimed to evaluate the diagnostic value of the MTPR and its relative change (relΔ_t__MTPR) over time. We analyzed structural MRI scans at a 1-year interval and defined the MTPR in patients with PSP and IPD as well as healthy controls (HC).

## Methods

### Participants

The study included 15 patients with probable or possible PSP, 15 patients with IPD as well as 15 HC. Trained movement disorders specialists (CJH; MS) confirmed clinical diagnoses of PSP and IPD, based on the NINDS diagnostic criteria [21]. Additionally, the MDS diagnostic criteria were applied, retrospectively to every patient [22]. Clinical records were reviewed, and groups were matched for age and disease duration (DD) at baseline (BL). The study was approved by the local ethics committee (study no. 2849). All participants gave prior written informed consent and all conducted study investigations were performed in accordance with the declaration of Helsinki [23].

### Magnetic resonance imaging and analysis

All participants underwent two MRI scans (BL and after 1 year ± 3 months) on a 3-T Siemens Tim Trio scanner (Siemens Healthcare GmbH, Erlangen, Germany). 3D T1-weighted images with 1.0 mm isotropic resolution were collected (MP RAGE, echo time = 2,98 ms, repetition time = 2300 ms, flip angle = 9°, acquisition matrix = 256 × 256, number of excitations = 1, field of view = 256 mm). MRI sequences were visually examined (JC; SK) to exclude relevant confounders such as movement artefacts or additional/differential diagnoses such as vascular lesions.

### Morphometric measurements

Morphometric measurements were manually assessed using 3D Slicer Version 4.10.2 (slicer.org). Midsagittal T1-weighted individual MRIs were used for the midbrain and pons measurements, using a simplified version of the methodology described by Massey et al. (2013) [17]. Two independent investigators (JC; SK) blinded to the diagnoses performed the analyses. Each investigator drew line measurements over pons and midbrain (maximal widths perpendicular to the visually estimated oblique superior–inferior axes) in a midsagittal slice to assess the respective area widths. In line with previous research, pons measurements did not include the pontine tegmentum and midbrain measurements did not include the collicular plate [17, 18]. The MTPR was calculated from the determined values dividing the width of the midbrain by the width of the pons for every individual. The relΔ_t__MTPR was defined as $\mathrm{rel}{\Delta }_{t}\_MTPR= \frac{({MTPR}_{1Y}- {MTPR}_{BL})}{{MTPR}_{BL}}$ with ${MTPR}_{BL}$ as baseline MTPR and ${MTPR}_{1Y}$ as MTPR after 1 year.

### Statistical analyses

All data were analyzed using IBM SPSS version 25.0 (IBM SPSS Statistics, Armonk, NY: IBM Corp.) and R version 3.6.3 (R Foundation for Statistical Computing, Vienna, Austria). Data were evaluated for normality using the Shapiro Wilk test. Parametric, non-parametric or Chi-squared tests were used for group comparisons, depending on the distribution of variables.

To assess inter-rater reliability (IRR) the measurements for midbrain and pons were analyzed using intraclass correlation coefficients (ICCs; single measures, consistency).

Between-group comparisons were performed with an unpaired t-test for DD at BL, Kruskal–Wallis analysis of variance (ANOVA) for age at BL and a Chi-squared test for gender distribution. A multivariate analysis of variance (MANOVA) was performed to investigate differences in midbrain, pons, and MTPR between groups and MRI time points. We calculated a combined parameter of MTPR_BL_ and relΔ_t__MTPR (MTPR_BL&Δt_) using predicted probability values from a binary logistic regression model. This new parameter was used as test variable in ROC analyses. Thus, it could be estimated whether the diagnostic accuracy of MTPR_BL_ can be significantly enhanced by adding relΔ_t__MTPR. Receiver operating characteristic curve (ROC) analyses were then performed to evaluate the predictive value of the MTPR, relΔ_t__MTPR, and MTPR_BL&Δt_ by computing the area under the curve (AUC; 95% CI). Diagnostic accuracy was determined in differentiating PSP *vs.* IPD *vs.* HC using the optimal cutoff value determined by ROC analysis with 95% confidence intervals. The cutoff was defined as the value resulting in the highest Youden Index. ROC curves were analyzed for significant differences using the roc.test command of the pROC package [24] in R (method = bootstrap, paired). We also performed a McNemar’s test, to evaluate statistical differences in specificity values. A *p* value < 0.05 was considered significant for all tests. For all statistical comparisons, post-hoc Bonferroni analyses were performed, to correct for multiple comparisons.

## Results

**Fig. 1:**
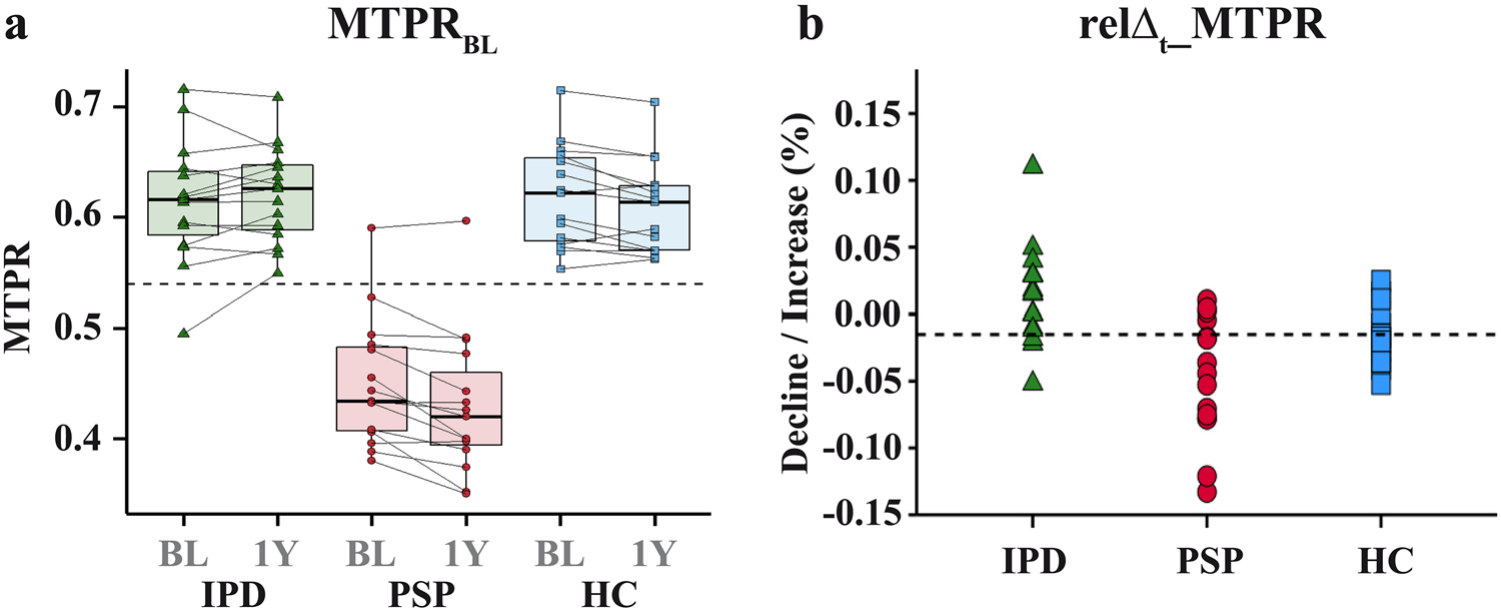
Main results for the observed groups. **a** MTPR for all groups at both time points; **b** relΔ_t__MTPR (decline/increase; %) for all groups. *MTPR* midbrain-to-pons ratio, *IPD* idiopathic Parkinson’s disease, *PSP* progressive supranuclear palsy, *HC* healthy controls, *BL* Baseline, *1Y* 1 year after baseline, *relΔt_MTPR* relative change, *MTPR*_*BL&Δt*_ combined parameter of MTPR_BL_ and relΔ_t__MTPR,  +  increase; − decrease

**Fig. 2:**
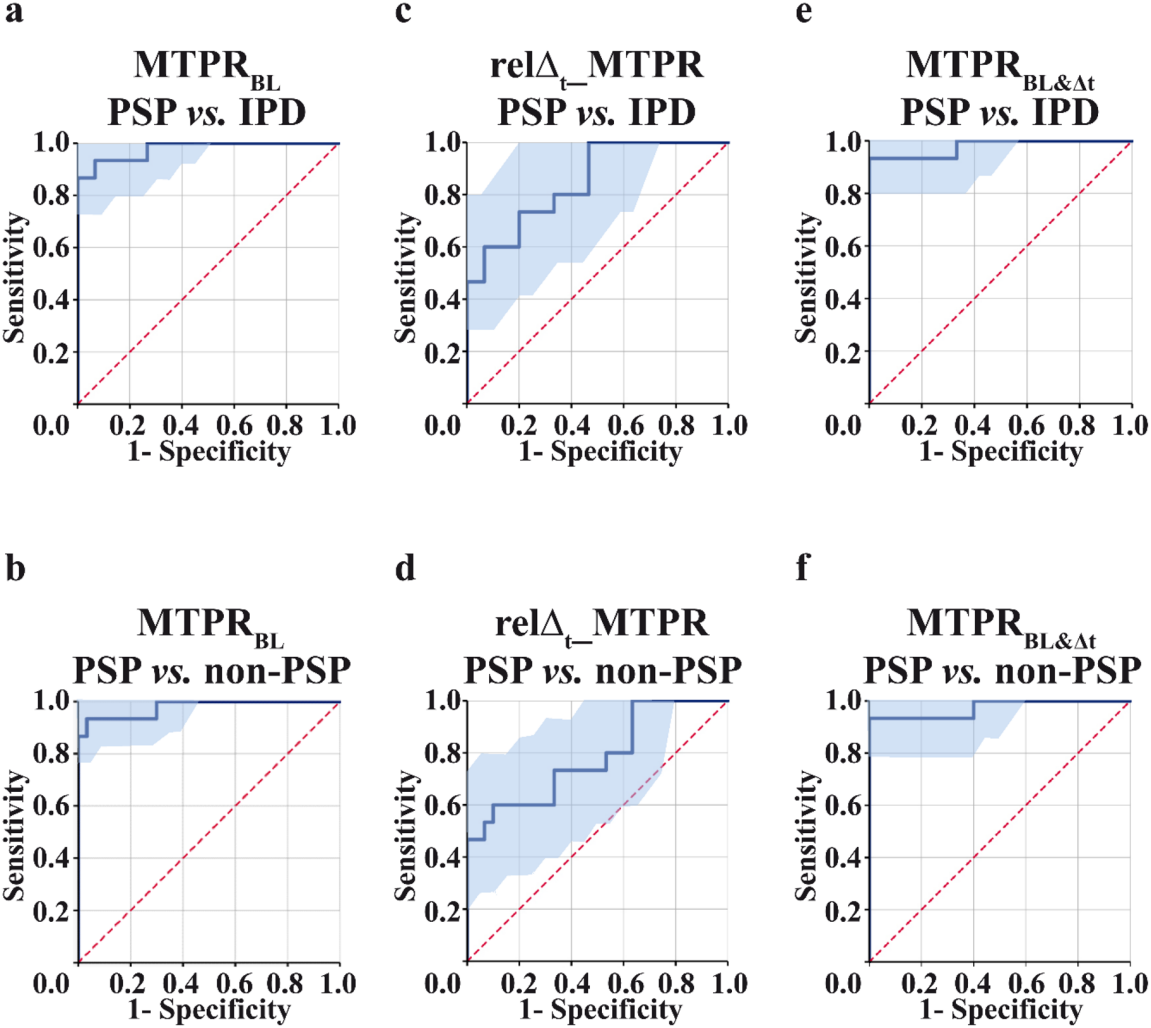
Main results of ROC analyses. ROC curve for MTPR_BL_ comparing **a** PSP vs. IPD and **b** PSP vs. non-PSP; **c** ROC curve for relΔ_t__MTPR comparing PSP vs. IPD and **d** PSP vs. non-PSP; **e** ROC curve for MTPR_BL&Δt_ comparing PSP vs. IPD and **f** PSP vs. non-PSP. *MTPR* midbrain-to-pons ratio, *IPD* idiopathic Parkinson’s disease *PSP* progressive supranuclear palsy, *HC* healthy controls, *BL* Baseline, *1Y* 1 year after baseline, *relΔt_MTPR* relative change, *MTPR*_*BL&Δt*_ combined parameter of MTPR_BL_ and relΔ_t__MTPR

Group characteristics and main findings for PSP, IPD, and HC are summarized in Table 1 and Figs. 1 and 2. The observed groups did not significantly differ in gender distribution, age, and DD at BL.

**Table 1:** Demographic and morphometric data of patients with PSP and IPD as well as HC

	IPD	BL vs. 1Y^c^	PSP	BL vs. 1Y^c^	HC	BL vs. 1Y^c^	PSP vs. non-PSP^c^	PSP vs. IPD*	PSP vs. HC*	HC vs. IPD*
N	15		15		15					
GD (m/f)^a^	9/6		10/5		8/7		n.s	n.s	n.s	n.s
Age_BL_ (years)^b,d^	64.00 ± 7.50		69.60 ± 3.91		64.47 ± 8.39		n.s	n.s	n.s	n.s
DD_BL_ (months)^b,c^	50.20 ± 31.34		63.13 ± 30.69		n.a		n.s	n.s	n.a	n.a
MW_BL_^b,c^	11.14 ± 0.79	n.s	8.17 ± 0.89	***p = 0.001***	10.99 ± 1.07	***p = 0.047***	***p < 0.001***	***p < 0.001***	***p < 0.001***	n.s
MW_1Y_^b,c^	11.09 ± 0.73		7.80 ± 1.08		10.84 ± 1.03		***p < 0.001***	***p < 0.001***	***p < 0.001***	n.s
PW_BL_^b,c^	18.23 ± 1.32	n.s	18.23 ± 1.42	n.s	17.77 ± 1.27	n.s	n.s	n.s	n.s	n.s
PW_1Y_^b,c^	17.92 ± 1.28		18.24 ± 1.47		17.82 ± 1.15		n.s	n.s	n.s	n.s
MTPR_BL_^b,c^	0.61 ± 0.05	n.s	0.45 ± 0.06	***p = 0.001***	0.62 ± 0.05	***p = 0.011***	***p < 0.001***	***p < 0.001***	***p < 0.001***	n.s
MTPR_1Y_^b,c^	0.62 ± 0.04		0.43 ± 0.06		0.61 ± 0.04		***p < 0.001***	***p < 0.001***	***p < 0.001***	n.s
relΔt_MTPR (%) ^b,c^	+ 1.29 ± 3.77		− 4.74 ± 4.48		− 1.58 ± 2.18		***p < 0.001***	***p < 0.001***	n.s	n.s
Cutoff MTPR (≤ 0.54)^a^	1 (6.67%)		14 (93.33%)		0 (0.00%)		***p < 0.001***	***p < 0.001***	***p < 0.001***	n.s
Sensitivity							93.33%	93.33%	93.33%	n.a
Specificity							96.67%	93.33%	100.00%	n.a
Predictive Accuracy							95.56%	93.33%	96.67%	n.a
Cutoff Dec. (≥ 1.45%)^a^	3 (20.00%)		11 (73.33%)		10 (66.67%)		n.s	***p = 0.009***	n.s	***p = 0.025***
Sensitivity							73.33%	73.33%	73.33%	n.a
Specificity							56.67%	80.00%	33.33%	n.a
Predictive Accuracy							62.22%	76.67%	53.33%	n.a
Cutoff MTPR_BL&Δt_ (≥ 0.52)^a^	0 (0.00%)		14 (93.33%)		0 (0.00%)		***p < 0.001***	***p < 0.001***	***p < 0.001***	n.s
Sensitivity							93.33%	93.33%	93.33%	n.a
Specificity							100.00%	100.00%	100.00%	n.a
Predictive Accuracy							97.77%	96.67%	96.67%	n.a

*IPD* idiopathic Parkinson’s disease, *PSP* progressive supranuclear palsy, *HC* healthy controls, *GD* gender distribution, *BL* baseline, *DD* disease duration, *MW* midbrain width, *1Y* 1 year after baseline, *PW* pons width, *MTPR* midbrain-to-pons ratio, *Dec* relative decline, *MTPR*_*BL&Δt*_ combined parameter of MTPR and relative change, *n.s.* not significant, *n.a.* not applicable,  +  increase; −  decrease

*Post-hoc, Bonferroni corrected. Significant *p* values are marked in bold

^a^Chi-squared test confirmed by Fisher’s exact test

^b^Values are given as mean ± standard deviation

^c^Parametric tests (One-way ANOVA; unpaired t-test; paired t-test; Repeated measures analysis of variance)

^d^Non-parametric tests (Kruskal–Wallis analysis of variance)

### Inter-rater reliability

ICCs revealed good IRR for pons measurements at BL (ICC = 0.87, *p* < 0.001), as well as after 1 year (ICC = 0.89, *p* < 0.001). Moreover, there was an excellent IRR for midbrain measurements at BL (ICC = 0.97, *p* < 0.001) and after 1 year (ICC = 0.98, *p* < 0.001). Given this high degree of consistency, averaged values from both raters for midbrain and pons were used to calculate MTPR_BL_ and MTPR_1Y_.

### Morphometric analyses

Most collected midbrain MRI measures revealed significant differences between PSP and non-PSP groups: PSP-patients had a smaller MTPR_BL_ (*M* = 0.45 ± 0.06) as well as MTPR_1Y_ (*M* = 0.43 ± 0.06) compared to both non-PSP groups (F (2, 42) = 66.87, *p* < 0.001; Fig. 1a). This difference was particularly driven by a smaller midbrain width (MW) at both time points in PSP-patients (F (2, 42) = 60.08, *p* < 0.001), whereas the MTPR and MW did not differ between IPD and HC. Furthermore, there was a significant decline in MTPR (t (14) = . 4.06, *p* = 0.001) over the 1-year period for the PSP group. The relΔ_t__MTPR was stronger in PSP compared to IPD (*p* < 0.001; Fig. 1b) with a mean decline of 4.7% in MTPR for PSP-patients. Conversely, the relΔ_t__MTPR did not differ between PSP (*M* = − 4.74 ± 4.48) and HC (*M* = − 1.58 ± 2.18). Here, the MTPR_1Y_ in HC was significantly smaller compared to the corresponding MTPR_BL_ (t (29) = 2.92, *p* = 0.011), which was not the case for patients with IPD. Pontine values did not differ between groups and time points (see Table 1 for statistical details).

### ROC analyses

ROC analyses confirmed excellent diagnostic accuracy for the MTPR_BL_ (AUC = 0.98, 95% CI 0.94–1.00, sensitivity = 93.33%, specificity = 93.33%, accuracy = 93.33%) when comparing PSP and IPD-patients (Fig. 2a), as well as PSP and both non-PSP groups (AUC = 0.98, 95% CI 0.94–1.00, cutoff ≤ 0.540, sensitivity = 93.33%, specificity = 96.67%, accuracy = 95.56%; Fig. 2b). Regarding relΔ_t__MTPR accuracy for distinguishing PSP from IPD was good (AUC = 0.85, 95% CI 0.72–0.98, cutoff ≥ 0.015, sensitivity = 73.33%, specificity = 80.00%, accuracy = 76.67%; Fig. 2c); Moderate diagnostic accuracy could be demonstrated for distinguishing PSP from non-PSP participants (AUC = 0.78, 95% CI 0.63–0.93, sensitivity = 73.33%, specificity = 56.67%, accuracy = 62.22%; Fig. 2d). There was also excellent diagnostic accuracy for MTPR_BL&Δt_ (AUC = 0.97, 95% CI 0.93–1.00, sensitivity = 93.33%, specificity = 100.00%, accuracy = 96.67%; Fig. 2e) when comparing PSP and IPD as well as for comparing PSP and non-PSP subjects (AUC = 0.98, 95% CI 0.92–1.00, OC ≥ 0.520, sensitivity = 93.33%, specificity = 100.00%, accuracy = 97.77%; Fig. 2f). There was no significant difference for the diagnostic accuracy of MTPR_BL&Δt_ and MTPR_BL_ (*D* = − 0.43, *p* = 0.67). Additionally, the specificity values did not differ significantly (*p* = 0.500; see Table 1 for all detailed values).

## Discussion

This is the first study to investigate the MTPR in a longitudinal setting to the best of our knowledge. Our cross-sectional results confirmed a lower MTPR_BL_ in PSP, when compared both to IPD and to HC; an MTPR_BL_ ≤ 0.54 was indicative of PSP. Longitudinal evidence revealed a distinct 1-year decline of MTPR in PSP-patients, representing a more pronounced midbrain atrophy rate compared to IPD. The combined MTPR_BL&Δt_ slighty improved the already high diagnostic accuracy of MTPR_BL_ and likewise improved the specificity to 100%; however, these improvements were not statistically significant.

Overall, MTPR values confirm previous findings suggesting an MTPR ≤ 0.52 as highly specific for PSP [17, 18]. Longitudinal results also tally with former research, as differentiation of PSP from IPD is based on studies demonstrating that PSP presents stronger and faster midbrain atrophy [25–27]. However, we were not able to discriminate between PSP and non-PSP groups solely by means of relΔ_t__MTPR as with the MTPR_BL_. Still, there was good predictive accuracy for distinguishing PSP and IPD only.

Most importantly, we found increased specificity values for the combined parameter MTPR_BL&Δt_. This is of particular clinical relevance considering that a high degree of specificity is very important for distinguishing between various forms of diseases [7]. However, a statistical comparison of specificity values did not reach significance. As the MTPR_BL_ provided already excellent specificity with 96.67%_,_ when comparing PSP and non-PSP groups, it is hard to improve specificity further in fact. However, with MTPR_BL&Δt_ specificity reached 100%.

With a mean DD of 63.1 months we investigated patients in rather progressed disease stages. This is particularly important considering that midbrain atrophy could also serve as PSP-specific preclinical marker in very early disease stages [28, 29]. Thus, it remains to be studied, whether the relΔ_t__MTPR—as indicator of midbrain atrophy rate—contributes better to diagnostic accuracy, earlier in the disease, i.e., when the overall MTPR has not yet reached PSP-specific values. Again, a larger patient cohort, e.g., from a future multi-centric study, would allow a more detailed analysis of effects of DD, age, and gender. While relΔ_t__MTPR was of limited diagnostic value in our patient cohort with advanced stages of the diseases, it might have a more valuable impact in early stages of PSP, where higher MTPR ratios can be expected.

Moreover, it has to be considered that atrophy rates might differ between different disease stages in PSP, as it was already reported for other neurodegenerative diseases [30]. Additionally, we did not include a quantitative measure of disease severity such as the Progressive Supranuclear Palsy Rating Scale or MDS-Unified Parkinson's Disease Rating Scale. This would have been helpful in estimating disease progression independently from DD.

Patients in this study were diagnosed clinically by expert evaluation; however, misdiagnoses cannot be excluded in the absence of post mortem verification. However, all diagnoses were based on valid diagnostic criteria [21] enhancing the reliability of the clinical diagnosis. Additionally, we have also attempted to retrospectively apply the MDS diagnostic criteria for PSP [22] to allow a more precise description of diagnosis. Four patients, were confirmed to be correctly classified as PSP, by post mortem diagnosis.

In our study, we also did not distinguish between PSP-subtypes, as reported patients were mostly diagnosed with PSP-Richardson`s syndrome. However, it cannot be excluded that distinct subtypes could also differ in atrophy rates, which again points out the importance of further analyses in larger samples.

Moreover, HC presented a decline in MTPR, too. Midbrain shrinkage has been found in healthy ageing previously and, therefore, may have contributed to MTPR reduction, as a significant decline of MW could also be observed [31, 32]. Still, midbrain atrophy is assumed to be more pronounced in PSP. Hence, it should be considered, if intersubject variability in PSP could also account for the results at hand.

The important new finding from the present work apart from the confirmation and replication of previous studies on this topic is that specificity values increase by adding the relΔ_t__MTPR to MTPR_BL._ This is of high clinical relevance in disease differentiation. Statistical comparison of specificity values did not reach significance, as the MTPR_BL_ already had very good values, which were difficult to improve. Still, we believe that the MTPR_BL&Δt_ can further substantiate diagnosis of PSP in follow-up examinations and serve as an additional biomarker of PSP-specific disease progression, which may of particular importance to reveal the efficacy of potential disease-modifying treatments. Finally, the current findings motivate larger patient studies including PSP subtypes and other forms of atypical Parkinsonian diseases to explore the full potential of MTPR and its change as diagnostic tool.

## Data Availability

The participants of this study did not agree for their data to be shared publicly. Hence, we are not able to offer them for further usage.
